# SpainUDP: The Spanish Undiagnosed Rare Diseases Program

**DOI:** 10.3390/ijerph15081746

**Published:** 2018-08-14

**Authors:** Estrella López-Martín, Beatriz Martínez-Delgado, Eva Bermejo-Sánchez, Javier Alonso, the SpainUDP Network, Manuel Posada

**Affiliations:** 1 Institute of Rare Diseases Research (IIER) & Centre for Biomedical Network Research on Rare Diseases (CIBERER), Instituto de Salud Carlos III, 28029 Madrid, Spain; eva.bermejo@isciii.es (E.B.-S.); mposada@isciii.es (M.P.); 2 Institute of Rare Diseases Research (IIER), Instituto de Salud Carlos III, 28029 Madrid, Spain; bmartinezd@isciii.es (B.M.-D.); fjalonso@isciii.es (J.A.)

**Keywords:** diagnosis delay, rare diseases, undiagnosed programs, standardized phenotype, phenotype ontologies, whole exome analysis, international data sharing

## Abstract

One of the IRDiRC goals for 2017–2027 is to achieve definitive diagnosis for rare undiagnosed diseases within one year, as delay in diagnosis remains one of the pending issues in the rare diseases field. The Spanish Undiagnosed Rare Diseases Program (SpainUDP) was created in response to this challenging scenario to cover patients’ needs and after seeing the success of the Undiagnosed Diseases Program (UDP) in the USA. SpainUDP offers a multidisciplinary approach to those patients who have long sought a diagnosis without any success. During the first phase of the protocol, undiagnosed cases are sent to SpainUDP by individual patients or families, patient organizations or hospitals. After careful analysis of phenotype, data from sequencing experiments (WES) is processed with a standard pipeline and detailed standardized phenotypic information (mapped to the Human Phenotype Ontology, HPO) is connected to genetic data. In addition, the participation of SpainUDP in international initiatives such as the European projects RD-Connect and Solve RD, the Undiagnosed Diseases Network International (UDNI), and the MatchMaker Exchange (MME) platform, allows the establishment of a global data sharing strategy across multiple projects submitting data to these international initiatives. From the official beginning of the program (at the end of 2015) until early 2018, 147 cases were accepted in SpainUDP. During this time, 37 cases (25%) dropped out the program due to several reasons. The remaining 110 cases are distributed as follows: phenotypic and genotypic (WES) characterization was finished in 30 cases, of which 20 (67%) were diagnosed; 21 cases are pending on variants’ validation by Sanger sequencing; in 25 cases, WES is ongoing and 34 cases are being studied for deep phenotypic characterization. In conclusion, SpainUDP aims to achieve a diagnosis following two recommendations of the IRDiRC: the patients’ diagnosis in as short a time as possible and the promotion of data sharing (especially genomic) at the international level.

## 1. Introduction

Rare diseases (RD) constitute a very heterogeneous group of clinical entities defined by low prevalence, high rates of mortality and long-term disability. Although substantial progress has been made toward identifying the genetic basis of rare diseases, the underlying etiologies for many of them remain undiscovered [1] and knowledge gaps continue to exist regarding genotype-phenotype correlation. In addition, the low number of cases in each geographical area and the difficulties for accessing data are still big obstacles for RD diagnosis and their research. Efforts of individual researchers continue to multiply while remaining largely “siloed”, with almost no information exchange [2], which makes it extremely complicated to extract and to use data for diagnostic purposes. On the other hand, difficulties to obtain the correct diagnosis in the presence of pathogenic variants in multiple genes of a specific patient, are particularly challenging [3].

Given this complex scenario, it is not surprising that clinical assessments and conventional genetic testing lead to a diagnosis in less than half of patients [4]. The complexity of these diseases determines that a high percentage of cases has to be referred to clinical centers with a high level of specialization and, finally, to undiagnosed RD programs (where they exist). These programs have developed systematic procedures based on an individualized and exhaustive study of patients by a multidisciplinary team. One of the pioneering programs for the management and resolution of undiagnosed RD cases was the National Institutes of Health (NIH) Undiagnosed Diseases Program (UDP), which was launched in 2008 in the United States to address the diagnosis of rare and previously unrecognized diseases. Based upon its success, some years later, the Undiagnosed Diseases Network (UDN) was created to extend the UDP model to other medical centers in the USA [5]. In addition, the Undiagnosed Diseases Network International (UDNI) was founded across several countries (Australia, Austria, Bulgaria, Canada, Hungary, India, Italy, Japan, South Korea, Spain, Sweden and the United States) to improve the rate of definitive diagnosis for persons living with undiagnosed conditions, at the international level [6]. Inspired by the UDN, the UDNI has built a consensus framework of principles, governance and best practices [7]. After the establishment of the NIH-UDP in the United States [5], other UDP initiatives have been launched in some European countries (Austria, Bulgaria, Hungary, Sweden, Italy and Spain) and in other continents (Japan, Korea, Australia and Canada) [8]. The emergence of undiagnosed RD programs has coincided with the expansion of next-generation sequencing technologies. These technologies have rapidly been adapted to clinical testing, and are radically changing the paradigm of clinical diagnosis. Although single-gene testing and gene panels still hold a great value for the diagnosis of many disorders, whole exome sequencing (WES) has shown success most often in patients who remain undiagnosed after the traditional approaches [9]. In parallel to the enormous advances in gene identification through WES, large-scale data sharing initiatives have been developed to recognize unrelated persons with pathogenic variant(s) in the same gene and an overlapping phenotype [1].

SpainUDP is the acronym for the “Spanish Undiagnosed Rare Diseases Program”, which has been implemented by the Institute of Rare Diseases Research (IIER) of the Instituto de Salud Carlos III (ISCIII), a governmental organization whose goal is to manage and to carry out biomedical research in Spain. It is also a financer member of IRDiRC. SpainUDP was presented for the first time in 2013 as a pilot program in response to the high number of undiagnosed cases’ consultations received directly in the IIER or through the Spanish Federation of Rare Diseases (FEDER) [10]. This program was presented during the first UDNI conference, held in Rome in 2014. In 2015, SpainUDP became fully established after signing an agreement between the IIER-ISCIII and the Foundation for Biomedical Research of the University Hospital Puerta de Hierro (UHPH), in Madrid, for supporting detailed clinical examination in very complex undiagnosed cases. Also, SpainUDP has been included within the “Strategy for the improvement of health care for patients with rare diseases in the Community of Madrid (2016–2020)” [11]. As most of the aforementioned undiagnosed RD programs, SpainUDP’s procedure is based on three main pillars: (i) an exhaustive and individualized study of each case, (ii) the use of next-generation sequencing techniques, and (iii) the establishment of data sharing systems at the international level.

Diagnosis delay remains one of the foremost issues in the rare diseases field, as reflected in one of the IRDiRC goals for 2017–2027, that encourages the RD community to achieve diagnosis of patients “*within one year if their disorder is known in the medical literature*”. In this regard, IRDiRC has made a recommendation which is stated as follows: “*all undiagnosable individuals should enter a globally coordinated diagnostic pipeline*” [12]. SpainUDP was created in response to this challenging scenario to offer a multidisciplinary approach to those patients who have long sought a diagnosis without any success in Spain. This paper aims to describe in detail the phases composing this program, the results obtained to date, as well as the data sharing strategy that implies the link of SpainUDP with local, national and international RD resources to maximize the possibility of finding a diagnosis.

## 2. Materials and Methods

The operating rules of SpainUDP (http://spainudp.isciii.es) are defined in a standardized protocol that establishes the criteria for patient selection, data collection, laboratory investigation and diagnosis. This protocol has been elaborated by the SpainUDP team, comprising several physicians from different medical specialties, geneticists, biologists, biochemists, bioinformaticians, and dysmorphologists who support the program with their knowledge and expertise. SpainUDP also counts on the support of the Spanish Rare Diseases Biobank (BioNER, http://bioner.isciii.es), the Spanish Rare Diseases Patient Registry (SpainRDR, https://spainrdr.isciii.es), and the Spanish Mutations Database (SpainMDB, http://spainmdb.isciii.es).

### 2.1. Access to the Program

Patients with undiagnosed diseases or their family members can apply to SpainUDP individually or through their patient organizations or hospitals. Regarding the latter, in addition to the UHPH, two more hospitals are collaborating closely with SpainUDP: The University Children Hospital Niño Jesús, Madrid, and the University Hospital Virgen del Rocío, Sevilla, since they are sharing complex pediatric undiagnosed cases in order to find a diagnosis by means of the SpainUDP approach. New agreements with other Spanish hospitals are under discussion and it is expected that they can be signed shortly, which will help to expand the action area of the program throughout the Spanish regions (although it is important to highlight that, currently, the lack of signed agreements is not a barrier for the access by patients residing in any Spanish province). Active involvement of patients’ organizations is important too. Thus, after many years collaborating in different aspects (also in undiagnosed cases), a closer collaboration with FEDER [10] has been established to provide help to undiagnosed patients and/or their relatives and to inform them about how to apply to SpainUDP. A similar role is being played by patient associations such as “Asociación Objetivo Diagnóstico” (Diagnosis Objective Association) and “Asociación D’Genes” (D’Genes Association) [13]. Moreover, individual patients asking to be included in the patient registry without a definitive diagnosis are invited to be assessed in SpainUDP.

### 2.2. Inclusion Criteria

The applicants have to meet the following inclusion criteria to enter the program:

- To show clinical signs and symptoms which are not clearly identifiable or attributable to a known rare disorder, despite extensive clinical investigations by specialists of the Spanish National Health System for a long time (diagnosis is still eluded).
- To provide the consent for registering in SpainRDR, for storing biological materials in BioNER, and for sharing de-identified clinical data and samples among undiagnosed RD experts from the UDNI [6] and other international networks.

Collaboration of their physicians and specialists is highly recommended, although it is not mandatory, since patient’s autonomy in decision-making always prevails over physicians’ opinions.

### 2.3. Deep Phenotyping

Cases are presented to the program by patient associations, any clinician and/or by either themselves or their families. Available clinical information, including photographs, imaging/radiography results and previously performed genetic studies are thus provided by both their families and physicians. The SpainUDP team collects and carefully reviews all the aforementioned information, requesting for lacking documents whenever necessary. After that, the team makes a recommendation to accept or reject the application. If the applicant is accepted, all data is stored and managed in a protected database. In parallel, a close collaboration is established with local healthcare services, to avoid unnecessary journeys, thus averting discomfort for the patients and their families. On the other hand, if necessary, a plan for up to a full week of inpatient clinical testing is organized with the UHPH specialists in order to perform all of the complementary tests needed to complete their deep phenotyping.

### 2.4. Genomic Analysis

Next generation sequencing techniques, mainly whole exome sequencing (WES), are performed by trio analysis (proband, father and mother) in the IIER. Trio-based WES is carried out by using Nextera Truseq Rapid Enrichment kit (Illumina, San Diego, CA, USA) and sequenced in a NextSeq500 Illumina platform with High Output Flow Cell 2 × 75 cartridges.

Raw genomic data from sequencing experiments of DNA samples of patients and relatives are processed through two different standardized protocols carried out in parallel in two centres: on the one hand, internally in the IIER and, on the other hand, following the RD-Connect validated analysis pipeline [14].

In the IIER, an in-house pipeline is applied to raw data. After initial preprocessing for quality control (fastqc v0.11.3 and trimmomatic v0.33), sequences are aligned to the reference genome “Human_g1k_v37” using BWA v0.7.12, and duplicate readings marked with Picard v1.140. Mean coverage obtained ranges from 40× to 90×, and percentages of the exome covered by at least 20 reads (20 ×) are higher than 86% in most cases. Realignment, recalibration of quality parameters and variant calling are performed jointly for the three individuals of the trios using GATK v3.4 (HaplotypeCaller). KGGSeq (v0.8) is used to filter variants according to different inheritance models and to annotate variants with information of genes, transcripts, functional predictors (SIFT, Polyphen2, LRT, Mutation Tester, CADD, FATHMM, PROVEAN and ClinVar), presence in population databases (MAF < 0.005), associated diseases and bibliography.

In parallel, raw genomic data is realigned and reprocessed through the RD-Connect validated analysis pipeline [14] and held in the centralized RD-Connect database [2,15]. The processed data is available for online analysis through a user-friendly interface to authorized users once all the required commitments are fulfilled. Moreover, phenotypic terms are extracted from clinical documents stored in the patient registry, mapped to HPO (Human Phenotype Ontology) [16] terms and uploaded into PhenoTips [17], a software tool available in the RD-Connect platform. RD-Connect genomics interface allows filtering and refining the results by mode of inheritance, population frequencies, in silico pathogenicity prediction tools and HPO codes [2].

Results of both analyses are manually reviewed by two independent researchers of SpainUDP with common criteria. After such review, a consensus is reached to select the candidate variants, which are confirmed by Sanger sequencing in all family members. Finally, various sources of information are consulted to build a report with a detailed review of the scientific evidence that associates each detected genetic variant with a specific disease or disorder.

### 2.5. Data Sharing

WES analysis is not limited to the identification of only known disease genes. On the contrary, also further approaches are accomplished to study candidate variants of unknown clinical significance, involving data sharing from undiagnosed patients in international platforms/networks. The RD-Connect platform enables a comparison of patient data from SpainUDP across multiple projects submitting data to this platform. Furthermore, it is possible to push data from PhenoTips to Phenome Central [18], a centralized repository that facilitates the matching of cases with similar clinical and genotypic profiles within larger shared international networks, such as the UDNI. Moreover, RD-Connect, Phenome Central and UDNI are participating in the Matchmaker Exchange (MME) [19], a federated platform that represents the largest effort to enable sharing specific case details to find similar cases. Since SpainUDP is contributing with its undiagnosed cases to these three international initiatives, it is indirectly connected to the remaining projects that belong to MME. If we do not find any matching after data sharing, functional analyses are planned and undertaken in collaboration with external experts or by ourselves.

### 2.6. Ethical Issues

Regarding ethical issues, our protocol includes the signing of several informed consents (IC) by patients admitted into SpainUDP: (i) IC for registering in SpainRDR, (ii) IC for storing biosamples in BioNER, (iii) IC for carrying out WES analysis. Recently, we have incorporated new statements to the last cited IC in order to comply ethical requirements from Phenotips [17] and Phenome Central [18] (including specific questions suggested by both resources). It was approved by the Ethics Committee for Research of the ISCIII on the 1 September 2017 with the project identification code CEI PI 54_2017. On the other hand, the IC for storing biosamples in BioNER and patient data in SpainRDR was approved by the same Ethics Committee on the 6th February 2017 with the project identification code CEI PI 74_2016.

## 3. Results

**Figure 1:**
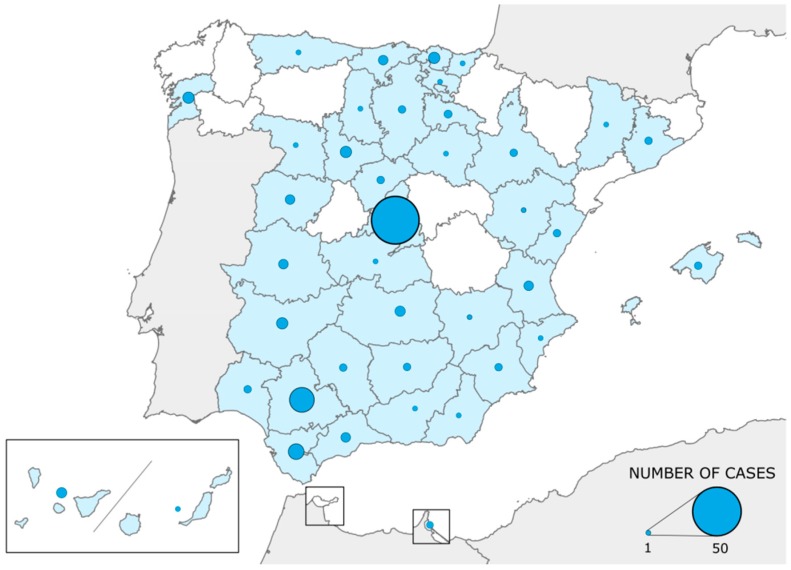
Geographical distribution of patients’ admissions in SpainUDP.

From the official launch of SpainUDP (in October 2015) to May 2018, 147 cases were accepted in SpainUDP. They were mainly pediatric, since 109 cases (74.1%) corresponded to patients younger than 18 years. Geographical distribution of the cases encompassed 40 (76.9%) of the 52 different Spanish provinces (Figure 1). Madrid (*n* = 50) and Seville (*n* = 14) were the provinces with the highest number of accepted cases.

**Figure 2:**
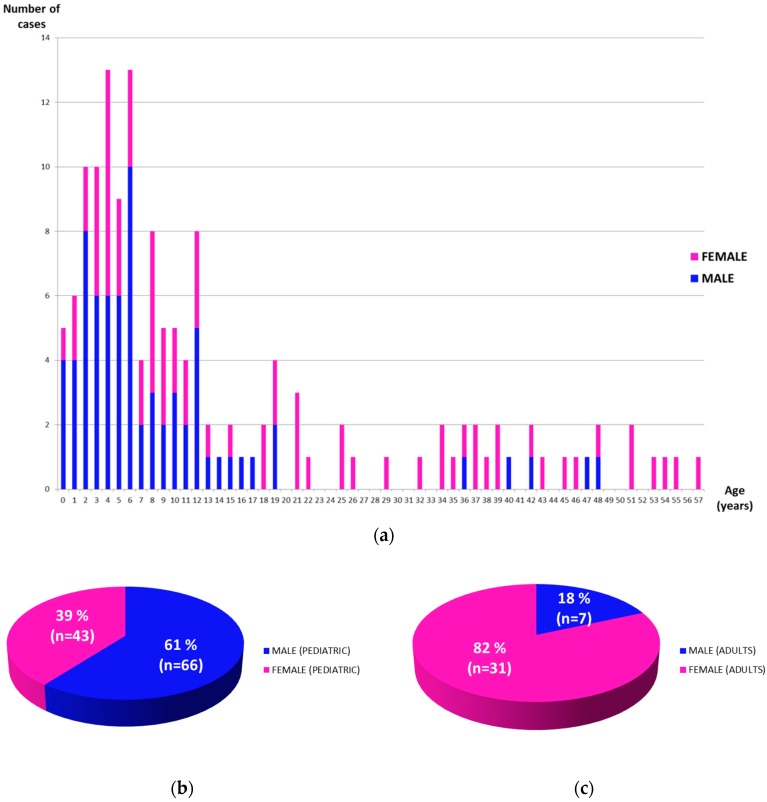
Distribution of SpainUDP patients by age and gender: (**a**) Bars graphic representing the distribution of patients by age and gender. (**b**) Pie chart representing the total distribution of pediatric patients by gender. (**c**) Pie chart representing the total distribution of adult patients by gender.

The distribution of patients by gender was very similar for both sexes (73 males and 74 females). However, the cases’ distribution by gender showed a different pattern depending on the age of patients (Figure 2a). Thus, taking into account only pediatric cases, there was a higher number of males (*n* = 66; 60.6%) than females (*n* = 43; 39.4%), with a sex-ratio of 1.54 (Figure 2b). This gender distribution reversed in adult patients as there was a higher number of females (*n* = 31; 81.6%) than males (*n* = 7; 18.4%) with a sex-ratio of 0.23 (Figure 2c).

**Figure 3:**
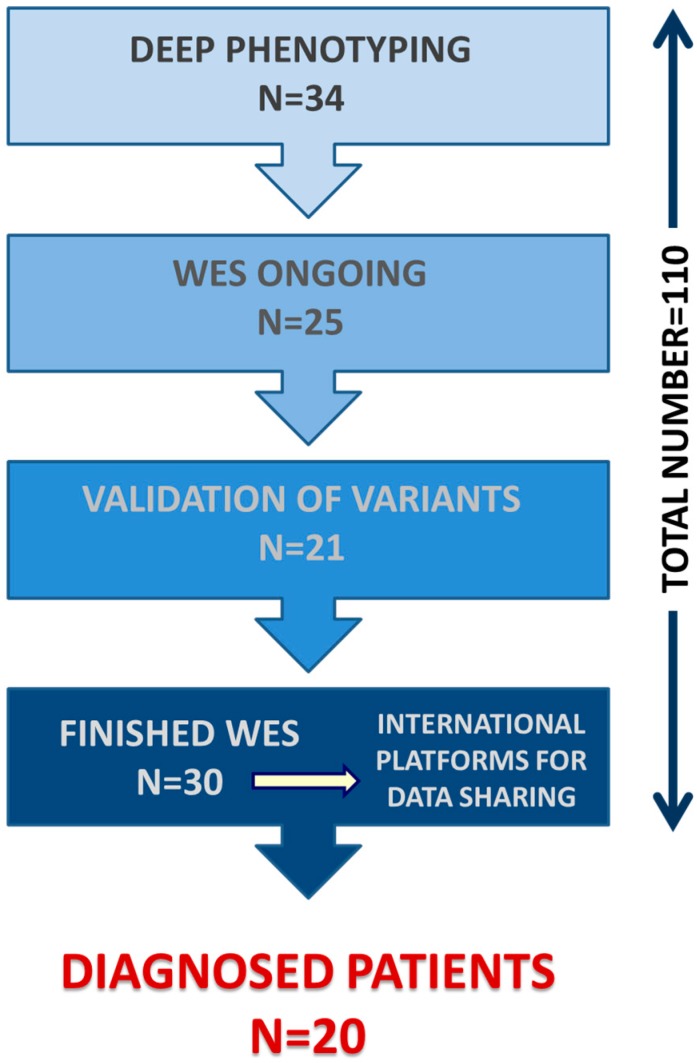
Main phases of SpainUDP and current number of patients within each one of them.

During this time period, 37 cases (25.2%) dropped out the program due to diverse reasons: they got their diagnosis independently of SpainUDP (*n* = 24); they had a common disease instead of a rare disease (*n* = 6); they decided to leave the program voluntarily (*n* = 3); the collaboration by the patients’ reference hospitals failed (*n* = 3); and they resided outside of Spain (*n* = 1). The remaining 110 cases are distributed as follows: phenotypic and genotypic (WES) characterization was finished in 30 cases, of which 20 (67%) were diagnosed; 21 cases are pending on variants’ validation by Sanger sequencing; in 25 cases, WES is ongoing; and the remaining 34 cases are being studied in depth for phenotypic characterization (Figure 3).

Some genetic testing with negative results were performed in all cases before their acceptance in SpainUDP. Prior negative genetic testing included: karyotype, CGH array, single-gene testing (MLPA, FISH, single-gene sequencing, etc.), study of mitochondrial DNA, methylation studies, gene panel and/or single WES in the proband. By far the most common phenotypic category of diagnosed cases was neurological disorders. In addition, these diseases could be considered as very rare conditions, since in most cases the prevalence was less than 1 per 1,000,000 inhabitants. Regarding genetic analyses, 75% of the detected causal variants corresponded to de novo mutations (mainly frameshift and stopgain variants). On the other hand, two patients (one male and one female) had the same genetic diagnosis (mental retardation autosomal dominant 32, associated to a mutation of the *KAT6A* gene). Table 1 displays a brief description of each one of the solved cases.

Table 2 shows age and gender data for the 20 diagnosed patients. Among these patients, 12 (60.0%) were male. However, the proportions of both diagnosed males (0.18, 12/66) and females (0.16, 7/43) were very similar and the difference between them was not statistically significant. The age at diagnosis ranged from 3.0 to 27.7 years (only one patient was over 18 years of age at the time of diagnosis) and the average age was 8.9 years. The mean time for diagnosis in SpainUDP was 13.7 months and ranged from 1.8 to 23.9 months.

After WES and despite great efforts and different strategies, ten cases remain unsolved to date:

- Case 1: a boy with a very complex phenotype including global developmental delay, osteoporosis and irregular hyperpigmentation.
- Case 2: a young woman with primary hyperparathyroidism, multiple bony cystic lesions and pathological fractures.
- Cases 3 and 4: two siblings with similar phenotypes characterized by global developmental delay, autistic features and hypotonia.
- Case 5: a boy with myoclonic epilepsy, autism spectrum disorder and frequent diarrheas.
- Case 6: a boy with severe lower limb amyotrophy, sensorimotor neuropathy and dysmorphic features.
- Case 7: a boy with a speech disorder and autistic behavior.
- Case 8: a girl with a systemic autoinflammatory disorder with a relevant digestive component (refractory to treatment).
- Case 9: a boy with remarkable dysmorphisms, such as prognathism, dental malocclusion and macrocephaly, and also absence crises, unexplained episodes of fever, intolerance to exercise and decreased muscle mass.
- Case 10: a boy who probably has two genetic diagnoses, as he has been diagnosed with epileptic encephalopathy early infantile type 4 (mutation of the *STXBP1* gene) which only explains part of his phenotype, but he also has bilateral sensorineural hearing impairment of unexplained origin.

Although WES analyses are currently finished for these cases, further investigations are being developed and it is expected that they lead to more diagnoses in the near future. Thus, we have candidate novel disease genes for 7/10 patients and they have been shared with other researchers through MME. By means of this platform, we have contacted with the owners of some other RD cases shared in this resource. Unfortunately, none of these cases showed any matching. On the other hand, we are performing expression studies of some of these candidate variants in our own laboratories and, also, we are carrying out some specialized studies in collaboration with external experts.

## 4. Discussion

Lack of screening tests and limited knowledge among health professionals about how to recognize the signs and symptoms of rare diseases lead to diagnostic delays [20]. A high rate of cases remains undiagnosed for many years and, as a consequence, these patients and their relatives do not know in advance neither the prognosis of their diseases, nor the possibilities to access to appropriate treatments. Programs aimed at studying such undiagnosed cases are currently growing and developing in many countries. In this regard, the UDNI is trying to validate the cooperation in this important field. The “Spanish Undiagnosed Rare Diseases Program” (SpainUDP), developed by the IIER-ISCIII, aims to solve some of these cases in Spain in close collaboration with the UDNI and other European projects such as RD-Connect and Solve-RD.

The majority of patients studied in SpainUDP were younger than 18 years. This is not surprising as the onset of rare diseases usually occurs during the first years of life [20]. It was also observed that the pediatric admissions into the program were more frequently males (60.6%) than females (39.4%). Similarly, 63.2% of the diagnosed pediatric patients were males, which reflects the higher number of males entering the program, since not significant differences in the diagnostic process were found in SpainUDP in relation to patients’ gender. A similar finding has been described in many birth defects and autism spectrum disorders, which are currently more common among males than females [21]. Several theories could explain the male-female discrepancy, such as a higher risk in males of being affected by X-linked recessive disorders and phenotypic differences in the presentation and/or severity between sexes. However, currently, we are not able to explain these differences found in the number of pediatric admissions and it will be necessary to check them with a higher number of cases. A higher admission of females (81.6%) was registered among adults. The reason for these sex differences is also unclear, but it could be partially explained by: (i) women tend to be affected by chronic diseases at younger ages than men [22] and, (ii) many menopausal women are more susceptible to varied symptoms because of changing hormone levels, which might be easily confounded with a rare disease. Based on our experience in SpainUDP, we consider that the diagnostic process in adults is frequently hard to manage as many interacting factors are contributing to the clinical status of patients. This conclusion is in accordance with the results described by Posey et al. [23], who observed a lower overall diagnostic rate in adults than in pediatric populations after diagnostic WES. Psychiatric disorders such as somatization and psychogenic disorders play also an important role at this point. Thus, sometimes psychiatric symptoms might be confounded by the patients, their families or even health care providers with symptoms produced by rare diseases. This is a challenging scenario, not so easy to manage by undiagnosed diseases programs due to the difficulties associated to these types of clinical diagnoses. Currently, three adult patients belonging to SpainUDP with suspicion of suffering somatization are being deeply studied to discard the possibility that they might have a rare disease (results not included in Table 1, since some tests are still pending). Similar findings were described in the article published by Gahl et al. [24], where six of 39 diagnosed patients were adults with fibromyalgia or common psychiatric disorders such as somatization, psychogenic movement disorder and psychogenic tic cough.

Regarding the geographical differences on patients’ admissions into SpainUDP, it was observed a high variability depending on patients’ residence provinces, being Madrid the province with the highest number of admitted patients. It is not an unexpected result as two of the three hospitals collaborating actively in SpainUDP (UHPH and University Children Hospital Niño Jesús) are located in Madrid and also this province has several millions of inhabitants. Patients who live in this province can freely choose the hospital to go to be attended by medical specialists, so they can be examined by the medical teams of the aforementioned hospitals without any administrative constraints. However, patients living outside Madrid need to request an official authorization by their health regional authorities to be transferred from their local hospital to the UHPH. Unfortunately, these types of administrative limitations generate some obstacles, which need to be avoided through a closer collaboration with their local specialists. Nonetheless, the administrative obstacles found for some patients could have been playing a role in the withdrawal of SpainUDP, since 29 (78.4%) of a total of 37 patients that dropped out the program lived outside of Madrid. This problem is an issue to which a special attention should be paid by our program and for which some administrative solution should be found to avoid frustration among patients who could benefit from this strategy and promote both equality and equity.

Deep phenotyping combined with exome sequencing allowed the diagnosis of 20 patients who were undiagnosed for a long time. We achieved a molecular diagnostic yield of 67% (20/30), which is quite higher than the diagnosis rates reported in other studies during the last years (25–37%) [25,26,27,28,29,30,31]. It is important to note that those studies were carried out in larger groups (hundreds or even thousands) of patients than ours (30 patients). On the other hand, some of the aforementioned studies also involved unselected consecutive referrals to clinical laboratories instead of studying patients who undergone specific inclusion criteria, as we routinely do in SpainUDP. Therefore, differences in the patients’ inclusion criteria could explain the discrepancy in diagnostic rates. Nevertheless, given our still low number of studied cases, our current diagnostic rate could change in the future. Our results (see Table 2) indicate that the average age at SpainUDP entry for these 20 patients was around 8 years and, since the onset for the majority of rare diseases occurs during the first years of life, we might assume that the average of the diagnosis delay in this group of patients before entering SpainUDP was approximately 8 years. Although the time period needed for diagnosis in SpainUDP was much shorter, with a mean value of 13.7 months (including the time for completion of the clinical documentation), we would like to indicate that only in 55.0% of the cases a diagnosis was achieved in less than one year, which would be desirable as it has been established by the IRDiRC [12]. On the other hand, after WES, 10 cases remain unsolved and their diagnosis delay will be then much higher than one year. Therefore, this is another problem to which pay attention and currently it is one of the most important challenges in SpainUDP, so we have to focus our efforts on reducing the time necessary for diagnosis.

The majority of the solved cases corresponds to children with pediatric neurological disorders. These disorders are often very complex and patients usually have wide and diverse phenotypes, such as intellectual disability, micro or macrocephaly, neurodevelopmental delay, feeding problems, epilepsy, hypotonia, brain structural anomalies, dysmorphic facial features, etc. As an example, in a study carried out by Yang et al. [25] for diagnostic evaluation of 2000 patients with suspected genetic disorders, patients were primarily pediatric (88%) with diverse clinical manifestations, most often including nervous system dysfunction. Both the complexity of these cases and the extremely low prevalence of their diagnosed diseases (see Table 1) are enough reasons that could explain why these patients had not been diagnosed in the National Health System and had to be referred to specialized programs such as SpainUDP. Also, despite the fact that some patients had been previously undergone gene panels according to their clinical manifestations, these did not include the gene finally identified in SpainUDP as disease causing. This once again demonstrates the utility of the strategy adopted in undiagnosed rare diseases programs like ours.

The use of the RD-Connect platform was very fruitful for the purposes of SpainUDP, since it enhanced the analyses, integration and sharing of very complex phenotypic and genotypic data managed in this program. However, unfortunately, the actions carried out in SpainUDP to date, to match our unsolved cases with similar phenotypic and genotypic profiles in platforms such as MME have failed. A truly optimization of this type of case-based matching on a global scale would require a major communication among researchers [1]. Although one of the strongest trends identified by IRDiRC is that “data sharing is the leitmotif, especially for genomics data” [32], and technical conditions are created to establish networks of patients data resources visible to clinicians and researchers regardless of the geographical location, there are still many open questions and future challenges that need to be solved. In order to get that researchers “feel free and sure to share data”, issues related to intellectual property rights would need to be assessed and handled in accordance with fundamental ethical rules and principles [33].

Current challenges for SpainUDP include the start-up of further actions for data sharing through the establishment of collaborations with other gene discovery-focused groups and the validation of variants of unknown significance through functional assays that demonstrate their pathogenicity. Thus, some pathogenic variants are being evaluated using cultured cells and animal models for functional assays that allow characterizing their mechanisms of pathogenicity. In this regard, currently, collaborations with some researchers are being established to accomplish these types of experimental studies. In addition to other European projects and strategies, Solve-RD and UDNI might be relevant tools to achieve these goals, since both initiatives pursue an integrated “beyond the exome” approach consisting of the use of sophisticated combined omics approaches.

## 5. Conclusions

In summary, the recognition of difficulty for diagnosis by the rare diseases’ community has triggered the emergence of special programs to investigate patients with undiagnosed disorders worldwide, such as SpainUDP. It aims to make appropriate diagnoses in rare diseases patients who still do not have a confirmed diagnosis, usually after a long time. At the same time, this multidisciplinary program, linked to a rare diseases research institute, aims to foster the discovery of new diseases through a translational approach. We also would like to emphasize the involvement of patients and their families with the diagnostic process carried out in SpainUDP, which has resulted in an extraordinary collaboration on their part, perhaps due to finding in this strategy a different approach to their “diagnostic odyssey”.

This article describes SpainUDP and synthesizes the first results obtained in its initial 2 years and how the collaboration with the UDNI and other international initiatives will open some hope for those patients who are still without a diagnosis. It also highlights the challenges and limitations in this long way for sharing experiences among rare diseases researchers.

## Figures and Tables

**Table 1:** Descriptive table of the cases diagnosed by SpainUDP.

Case	Molecular Testing before Entry in SpainUDP	Candidate Gene (Acronym & OMIM Number)	Mutation Effect	Mutation Type	Diagnosis (Disease Name & OMIM Number)	Inheritance ^1^	Prevalence ^2^
1	Karyotype, CGH array, single-gene testing & methylation study	*MEF2C* (OMIM 600662)	Stopgain	De novo	Mental retardation, autosomal dominant 20 (OMIM 613443)	AD	<1/1,000,000
2	Karyotype & single-gene testing	*VPS13B* (OMIM 607817)	Stopgain/Splicing	Compound heterozygous	Cohen syndrome (OMIM 216550)	AR	Unknown ^3^
3	Single-gene testing	*SLC52A2* (OMIM 607882)	Missense	Homozygous	Brown-Vialetto-Van Laere syndrome 2 (OMIM 607882)	AR	<1/1,000,000
4	Karyotype, CGH array & single-gene testing	*AUTS2* (OMIM 607270)	Frameshift	De novo	Mental retardation, autosomal dominant 26 (OMIM 615834)	AD	<1/1,000,000
5	Karyotype, CGH array & single-gene testing	*CTNNB1* (OMIM 116806)	Frameshift	De novo	Mental retardation, autosomal dominant 19 (OMIM 615075)	AD	<1/1,000,000
6	Karyotype & CGH array	*EHMT1* (OMIM 607001)	Stopgain	De novo	Kleefstra syndrome 1 (OMIM 610253)	AD	<1/1,000,000
7	CGH array	*SCN2A* (OMIM 182390)	Frameshift	De novo	Epileptic encephalopathy, early infantile, 11 (OMIM 613721)	AD	Unknown
8	Karyotype, CGH array & single-gene testing	*KMT2D* (OMIM 602113)	Frameshift	De novo	Kabuki syndrome 1 (OMIM 147920)	AD	1–9/100,000
9	Karyotype & single-gene testing	*PIK3R1* (OMIM 171833)	Missense	De novo	Short syndrome (OMIM 269880)	AD	<1/1,000,000
10	Karyotype, CGH array & single-gene testing	*DDX3X* (OMIM 300160)	Frameshift	De novo	Mental retardation, X-linked 102 (OMIM 300958)	XLD, XLR	<1/1,000,000
11	CGH array and mitochondrial DNA study	*PNPT1* (OMIM 610316)	Stopgain/Missense	Compound heterozygous	Combined oxidative phosphorylation deficiency 13 (OMIM 614932)	AR	<1/1,000,000
12	Karyotype, CGH array & single-gene testing	*KAT6A* (OMIM 601408)	Frameshift	De novo	Mental retardation, autosomal dominant 32 (OMIM 616268)	AD	<1/1,000,000
13	Single-gene testing	*SLC6A1* (OMIM 137165)	Frameshift	De novo	Myoclonic-atonic epilepsy (OMIM 616421)	AD	Unknown
14	Karyotype, CGH array & single-gene testing	*SYNGAP1* (OMIM 603384)	Frameshift	De novo	Mental retardation, autosomal dominant 5 (OMIM 612621)	AD	Unknown
15	Karyotype, CGH array & mitochondrial DNA study	*SLC9A6* (OMIM 300231)	Frameshift	De novo	Mental retardation, X-linked, syndromic, Christianson type (OMIM 300243)	XLD	<1/1,000,000
16	CGH array & single-gene testing	*KCNQ2* (OMIM 602235)	Missense	De novo	Epileptic encephalopathy, early infantile, 7 (OMIM 613720) & Seizures, benign familial neonatal, 1 (OMIM 121200)	AD	Unknown
17	Single-gene testing & gene panel	*NKX2-1* (OMIM 600635)	Frameshift	De novo	Choreoathetosis and congenital hypothyroidism with or without pulmonary dysfunction (OMIM 610978)	AD	<1/1,000,000
18	Single-gene testing, mitochondrial DNA study, gene panel & single-WES in proband	*VPS13D* (OMIM 608877)	Missense/Missense	Compound heterozygous	Movement disorder with ataxia and spasticity(no OMIM code yet)	AR	Unknown
19	Karyotype, CGH array & single-gene testing	*FRMPD4* (OMIM 300838)	Missense	Hemyzygous	Mental retardation, X-linked 104 (OMIM 300983)	XLR	Unknown
20	Karyotype, CGH array, single-gene testing & gene panel	*KAT6A* (OMIM 601408)	Missense	De novo	Mental retardation, autosomal dominant 32 (OMIM 616268)	AD	<1/1,000,000

^1^ AD = autosomal dominant, AR = autosomal recessive, XLD = X-linked dominant & XLR = X-linked recessive. ^2^ Prevalence data extracted from Orphanet database (accessed on 17th May 2018). ^3^ Prevalence is unknown and only 200 have been described to date worldwide.

**Table 2:** Gender and age statistics for the cases diagnosed by SpainUDP.

Case	Gender	Age at SpainUDP Entry (Years)	Age at Diagnosis (Years)	Time to Diagnosis (Months)
1	female	5.3	6.8	18.3
2	female	3.1	3.2	1.8
3	female	26.8	27.7	10.6
4	male	2.2	3.0	9.4
5	male	4.6	5.3	8.3
6	female	11.1	12.5	16.6
7	male	2.9	4.1	13.5
8	male	6.0	7.2	14.4
9	male	8.5	9.9	16.0
10	female	9.9	11.4	18.7
11	male	1.5	3.0	18.1
12	female	6.4	8.1	19.5
13	female	6.3	7.3	11.4
14	male	14.0	14.9	11.2
15	male	7.2	8.1	10.5
16	male	6.1	6.9	10.1
17	male	12.7	13.6	10.8
18	female	8.5	9.7	13.5
19	male	5.6	7.6	23.9
20	male	6.6	8.1	17.8
	***Mean ± SD***	7.8 ± 5.6	8.9 ± 5.6	13.7 ± 5.0
	***Median***	6.4	7.8	13.5
	***Minimum value***	1.5	3.0	1.8
	***Maximum value***	26.8	27.7	23.9
	***10th percentile***	2.3	3.1	8.4
	***90th percentile***	13.9	14.8	19.4

## Appendix A. SpainUDP Network

**Name**	**Institution**	**Department**	**City**
Isabel Cuesta	Instituto de Salud Carlos III	Bioinformatics	Majadahonda (Madrid)
Sara Monzón	Instituto de Salud Carlos III	Bioinformatics	Majadahonda (Madrid)
Julián Lara	University Hospital Puerta de Hierro	Pediatrics	Majadahonda (Madrid)
Rosario Cazorla	University Hospital Puerta de Hierro	Pediatrics	Majadahonda (Madrid)
Gemma Iglesias	University Hospital Puerta de Hierro	Pediatrics	Majadahonda (Madrid)
Enriqueta Román	University Hospital Puerta de Hierro	Pediatrics	Majadahonda (Madrid)
Purificación Ros	University Hospital Puerta de Hierro	Pediatrics	Majadahonda (Madrid)
Pablo Tutor	University Hospital Puerta de Hierro	Internal Medicine	Majadahonda (Madrid)
Susana Mellor	University Hospital Puerta de Hierro	Internal Medicine	Majadahonda (Madrid)
Carlos Jiménez	University Hospital Puerta de Hierro	Neurology	Majadahonda (Madrid)
Mª José Cabrejas	University Hospital Puerta de Hierro	Clinical Biochemistry	Majadahonda (Madrid)
Emiliano González	University Hospital Puerta de Hierro	Clinical Biochemistry	Majadahonda (Madrid)
Luis González	University Children Hospital Niño Jesús	Pediatrics	Madrid
Marcos Madruga	University Hospital Virgen del Rocío	Pediatrics	Sevilla
